# Establishment and maintenance of DNA methylation in nematode feeding sites

**DOI:** 10.3389/fpls.2022.1111623

**Published:** 2023-01-10

**Authors:** Morgan Bennett, Tracy E. Hawk, Valeria S. Lopes-Caitar, Nicole Adams, J. Hollis Rice, Tarek Hewezi

**Affiliations:** Department of Plant Sciences, University of Tennessee, Knoxville, TN, United States

**Keywords:** Arabidopsis, *Heterodera schachtii*, *Meloidogyne incognita*, DNA methylation, active *DNA demethylation*, promoter activity, syncytium, gall

## Abstract

A growing body of evidence indicates that epigenetic mechanisms, particularly DNA methylation, play key regulatory roles in plant-nematode interactions. Nevertheless, the transcriptional activity of key genes mediating DNA methylation and active demethylation in the nematode feeding sites remains largely unknown. Here, we profiled the promoter activity of 12 genes involved in maintenance and *de novo* establishment of DNA methylation and active demethylation in the syncytia and galls induced respectively by the cyst nematode *Heterodera schachtii* and the root-knot nematode *Meloidogyne incognita* in Arabidopsis roots. The promoter activity assays revealed that expression of the CG-context *methyltransferases* is restricted to feeding site formation and development stages. C*hromomethylase1* (*CMT1*), *CMT2*, and *CMT3* and *Domains Rearranged Methyltransferase2* (*DRM2*) and *DRM3*, which mediate non-CG methylation, showed similar and distinct expression patterns in the syncytia and galls at various time points. Notably, the promoters of various DNA demethylases were more active in galls as compared with the syncytia, particularly during the early stage of infection. Mutants impaired in CG or CHH methylation similarly enhanced plant susceptibility to *H. schachtii* and *M. incognita*, whereas mutants impaired in CHG methylation reduced plant susceptibility only to *M. incognita.* Interestingly, hypermethylated mutants defective in active DNA demethylation exhibited contrasting responses to infection by *H. schachtii* and *M. incognita*, a finding most likely associated with differential regulation of defense-related genes in these mutants upon nematode infection. Our results point to methylation-dependent mechanisms regulating plant responses to infection by cyst and root-knot nematodes.

## Introduction

DNA methylation and demethylation pathways regulate many aspects of plant development and stress responses through reversible, non-genetic modification of cytosine to 5-methylcytosine (5mC) (Fransz and De Jong, 2002; Henderson and Jacobsen, 2007; Zilberman et al., 2007; Zhang et al., 2010; Matzke and Mosher, 2014; Du et al., 2015; López Sánchez et al., 2016; Hewezi et al., 2018; Hewezi, 2020). In plants, this occurs in the CG, CHG, and CHH sequence contexts (where H is any nucleotide except G) (Lister et al., 2008). The *de novo* formation of 5mC is catalyzed by DNA methyltransferases DOMAINS REARRANGED METHYLTRANSFERASE2 (DRM2) and DRM3 through the RNA-directed DNA methylation (RdDM) pathway (Cao and Jacobsen, 2002a; Cao and Jacobsen, 2002b; Cao et al., 2003; Henderson et al., 2010). Following DNA replication and cell division, DNA methylation is maintained in a sequence context-specific manner (Saze et al., 2003). CG methylation is maintained mainly by METHYLTRANSFERASE1 (MET1). MET1 paralogs, MET2b and MET3, also contribute to CG maintenance but to a much lesser extent (Kankel et al., 2003; Quadrana et al., 2016). CHROMOMETHYLASE1 (CMT1), CMT2, CMT3, and the RdDM pathway are responsible for maintaining non-CG methylation (Henderson et al., 2010; Law and Jacobsen, 2010; Costa-Nunes et al., 2014; Stroud et al., 2014). 5mC can be actively removed and replaced by cytosine *via* the base excision repair process mediated by the paralogous DNA demethylases REPRESSOR OF SILENCING 1 (ROS1), DEMETER (DME), DME-LIKE2 (DML2) and DML3 (Choi et al., 2002; Gong et al., 2002; Agius et al., 2006; Morales-Ruiz et al., 2006; Penterman et al., 2007b; Gehring et al., 2009; Zhang and Zhu, 2012). Together, DNA methylation and demethylation fine-tune genome-wide methylation levels and subsequent transcriptional reprogramming (Gong et al., 2002; Penterman et al., 2007a; Penterman et al., 2007b; Zhu et al., 2007; Ortega-Galisteo et al., 2008; Lei et al., 2015; Bennett et al., 2022).

Recent studies have provided intriguing evidence for the involvement of DNA methylation and active demethylation pathways in modulating defense responses against various phytopathogens (Hewezi et al., 2018). Genome-wide methylation profiling of Arabidopsis leaves inoculated with *Pseudomonas syringae* pv. *tomato* DC3000 (Pst) revealed widespread changes in plant methylomes, particularly in gene-rich regions (Dowen et al., 2012). Pst-induced DNA methylation changes were associated with differential transcript abundances of a significant number of stress-responsive genes (Dowen et al., 2012). Similarly, infection of canola (*Brassica napus*) by the fungal pathogen *Leptosphaeria maculans* triggered differential DNA methylation in the promoters of thousands of genes both in resistant and susceptible cultivars, of which numerous genes are associated with defense responses (Tirnaz et al., 2020). The correlation between genome-wide DNA methylation patterns and gene expression levels were also reported in rice and tobacco in response to infection by rice Black Streaked Dwarf Virus and Cucumber Mosaic Virus, respectively (Wang et al., 2018; Li et al., 2020).

Changes in DNA methylation levels seem to impact plant interactions with fungal pathogens and oomycetes. For example, hypomethylated mutants defective in the establishment of DNA methylation increased plant resistance to *Hyaloperonospora arabidopsidis* (*Hpa*), whereas hypermethylated mutants impaired in active DNA demethylation increased plant susceptibility to both *Hpa* and *Fusarium oxysporum* (Le et al., 2014; Lopez Sanchez et al., 2016). Gene expression analyses of these mutants provided strong evidence for the involvement of DNA methylation in the regulation of numerous genes with defense- and stress-related functions. Notably, modulation of defense- and stress-responsive genes in hyper- or hypomethylated mutants showed opposite responses to various phytopathogens (López Sánchez et al., 2016).

Cyst nematodes (*Globodera* and *Heterodera* spp.) and root-knot nematodes (*Meloidogyne* spp.) are sedentary obligate biotrophs that infect the root systems of a broad-spectrum of host plants, including the model plant Arabidopsis. Nematode parasitism of host plants is characterized by the formation of syncytia and giant-cells as permanent feeding sites for cyst and root-knot nematodes, respectively. The formation of giant-cells by root-knot nematodes is accompanied by the formation of galls at the site of infection as a result of increasing cell division of neighboring cells. The parasitic second-stage juveniles (J2s) feed from these metabolically hyperactive feeding cells and develop into adult females and complete the life cycle. Several experimental evidences indicate that epigenetic mechanisms, particularly DNA methylation, play important roles in plant-nematode interactions (Hewezi, 2020). For example, mutants partially impaired in non-GC DNA methylation exhibited reduced susceptibility to cyst and root-knot nematodes (Hewezi et al, 2018; Ruiz-Ferrer et al., 2018; Atighi et al., 2021). Genome-wide DNA methylation analysis of soybean roots exposed to soybean cyst nematode (SCN, *Heterodera glycines*) revealed widespread DNA hypomethylation in the promoter and transcribed regions of a substantial number of genes (Rambani et al., 2015) that were previously reported to be differentially expressed in the syncytium (Ithal et al., 2007; Klink et al., 2009, Klink et al., 2010; Kandoth et al., 2011). Similarly, beet cyst nematode (BCN, *Heterodera schachtii*) was found to induce considerable increases in hypomethylation levels of protein-coding genes and transposable elements (TEs) (Hewezi et al., 2017; Piya et al., 2017). Further analysis indicated that BCN-induced DNA methylation changes may directly impact the transcript abundance of more than one-fourth of differentially expressed genes in syncytium (Hewezi et al., 2017). Methylome analysis of two near isogenic lines of soybean differing in their response to SCN revealed distinct and specific methylation profiles over protein-coding and miRNA genes as well as TEs (Rambani et al., 2020a; Rambani et al., 2020b). While the susceptible line exhibited global hypomethylation patterns, the resistant line showed global hypermethylation (Rambani et al., 2020a; Rambani et al., 2020b). Global DNA hypomethylation patterns, predominantly in the CHH context, were also observed in early developing galls induced by the root-knot nematode *Meloidogyne graminicola* in rice (Atighi et al., 2021). In contrast, it has been recently shown that early developing galls formed by *Meloidogyne javanica* have undergone hypermethylation particularly in the CHG context (Silva et al., 2022).

The biological significance of DNA methylation changes induced by parasitic nematodes was established using various functional assays of overexpression and mutant analyses (Hewezi et al., 2017; Rambani et al., 2020a; Rambani et al., 2020b; Atighi et al., 2021; Silva et al., 2022). However, the transcriptional activity of key genes involved in DNA methylation and active demethylation in nematode feeding sites remains mostly unknown. We addressed this issue by profiling the spatio-temporal expression patterns of genes involved in maintenance and *de novo* establishment of DNA methylation as well as those involved in active DNA demethylation in the syncytia and galls induced respectively by *H. schachtii* and *M. incognita* in Arabidopsis roots. Also, mutants of these genes were assayed for susceptibility to *H. schachtii* and *M. incognita.* Our analyses revealed a key role of DNA methylation and active demethylation in mediating plant susceptibility to two evolutionary distant nematode species.

## Materials and methods

### Plant materials and growth conditions

All transgenic Arabidopsis (*Arabidopsis thaliana*) lines expressing the GUS reporter gene under the control of various DNA methylation and demethylation related-genes were generated in the Columbia‐0 (Col‐0) background (Bennett et al, 2021). All mutant lines were generated in the Col‐0) background, expect *ros1* (*CS66099*) is in the C24 background. The mutant lines *cmt3-7* (*CS6365*, Lindroth et al., 2001), *drm2-2* (*CS16386*, Cao and Jacobsen, 2002a), and *ros1-1* (*CS66099*, Gong et al., 2002) have been previously characterized. T-DNA insertional mutants for *met2* (*SALK_102231C* and *SALK_093835C*), *met3* (*SALK_024049C* and *SALK_099592C*), *cmt1* (*SALK_138685C* and *SALK_030404C*), *cmt2* (*SALK_012874C* and *CS879822*), *cmt3* (*CS6365*) *drm2* (*CS16386* and *SALK_129477C*), *drm3* (*SALK_136439C* and *SALK_024820C*), *ros1* (*CS66099* and *SALK_045303C*), *dml2* (*SALK_131712C*) and *dml3* (*SALK_056440C*) were obtained from Arabidopsis Biological Resource Center (ABRC) (**Supplementary Figure 1A**). mRNA expression levels of these genes were quantified in uncharacterized mutant lines and the corresponding wild-type plants using reverse transcription quantitative PCR (RT-qPCR) (**Supplementary Figure 1B**). Ten-day-old plants grown in plates containing MS medium were used for RNA extraction and quantification of gene expression levels as described below. Length of the main root of 15 two-week-old plants per line was measured, and statistically significant differences from wild-type plants were calculated using *t* tests (*P* < 0.05). In addition, shoot and root phenotypes of all mutant lines along with the corresponding wild-type plants were assessed in four-week-old soil-grown plants.

### RNA isolation and RT-qPCR

Total RNA was isolated from mutant lines and the corresponding wild-type plants using the method previously described by Verwoerd et al. (1989), and then treated with DNase I (Invitrogen). DNase-treated RNA samples were diluted to a concentration of 50 ng/µL and used as a template for RT-qPCR reactions. The reactions were performed using Verso 1-step RT-qPCR (Thermo Fisher Scientific) in QuantStudio 6 Flex (Applied Biosystems) under the following conditions: 50°C for 15 min, 95°C for 15 min, and 40 cycles of 95°C for 15 s and 55°C for 30 s. Gene expression levels were quantified using 3 biological and 2 technical replicates. Quantification of expression levels was conducted using the 2^-ΔΔCT^ method (Livak and Schmittgen, 2001). *Actin8* (*AT1G49240*) and *PROTEIN PHOSPHATASE 2A SUBUNIT A3* (*PP2AA3*, *AT1G13320*) were used as internal control to normalize gene expression levels (Piya et al., 2019; Piya et al., 2020; Bennett et al., 2021). ΔCT values determined using *Actin8* and *PP2AA3* were very similar. Primers used for RT-qPCR assays were provided in **Supplementary Table 1.**

### Histochemical analysis of GUS activity

Seeds of the transgenic GUS reporter lines were sterilized using a 2.8% bleach solution for 5 minutes followed by several washes with sterilized distilled water. The sterilized seeds were randomly planted in culture plates (BD Biosciences) containing modified Knop’s medium solidified with 0.8% Daishin agar (Brunschwig Chemie). Ten-day-old plants were inoculated with about 100 surface-sterilized J2 of *Heterodera schachtii* or *Meloidogyne incognita.* Histochemical GUS staining was performed at 3, 7, 10, and 14 days post inoculation (dpi) for *H. schachtii* and 4, 7, and 14 dpi for *M. incognita*, according to Jefferson et al. (1987). Samples were incubated with the X-Gluc substrate (5-bromo-4-chloro-3-indolyl-beta-D-glucuronic acid, cyclohexylammonium salt) at 37°C and checked every 30 minutes for colorimetric changes. After staining, the GUS solution was replaced with 70% ethanol to terminate the reaction and samples were stored in ethanol at room temperature. At least two independent transgenic lines were assayed for each reporter line, with at least 24 replicated plants per line. Nematode-inoculated roots were imaged immediately following GUS staining. Images were captured using an EVOS M7000 microscope with a 4x lens for 3 and 4 dpi images and a 2x lens for all other time points.

### Nematode susceptibility assays

*H. schachtii* infection assays were determined in 12-well plates containing modified Knop’s medium (Sijmons et al., 1991) solidified with 0.8% Daishin agar. Seeds of the mutants and corresponding wild type were surface-sterilized and planted in a randomized block design. Ten days after planting, each seedling was inoculated with approximately 200 surface-sterilized J2 of *H. schachtii*. Following inoculation, the plates were incubated in the dark at 24°C for 3 days to facilitate nematode infection. The plants were then grown in 16-h-light/8-h-dark cycles at 24°C for three weeks before counting the number of J4 females per root system. Two independent experiments were performed with 20 replicates per genotype for each mutant and their corresponding wild-type controls.

For *M. incognita* infection assays, seeds of the mutants and wild type were surface-sterilized and planted on modified Knop’s-containing plates. Ten-day-old seedlings were then transplanted to pots containing steam‐sterilized sand mixed with top soil at a 3 to 1 ratio and organized in a randomized complete block design. A week after transplantation, each plant was inoculated with approximately 500 J2 *M. incognita* and grown in 16-h-light/8-h-dark cycles at 26°C. Whole root systems were collected five weeks after inoculation and the number of galls per root system was counted using light microscope. Statistically significant differences between the mutant lines and wild-type controls were calculated using a modified *t* test in the statistical software package SAS, where *P* values less than 5% were considered statistically significant.

## Results

### Promoter activity of DNA methyltransferases and demethylases in the syncytia induced by the beet cyst nematode *Heterodera schachtii*

**Figure 1:**
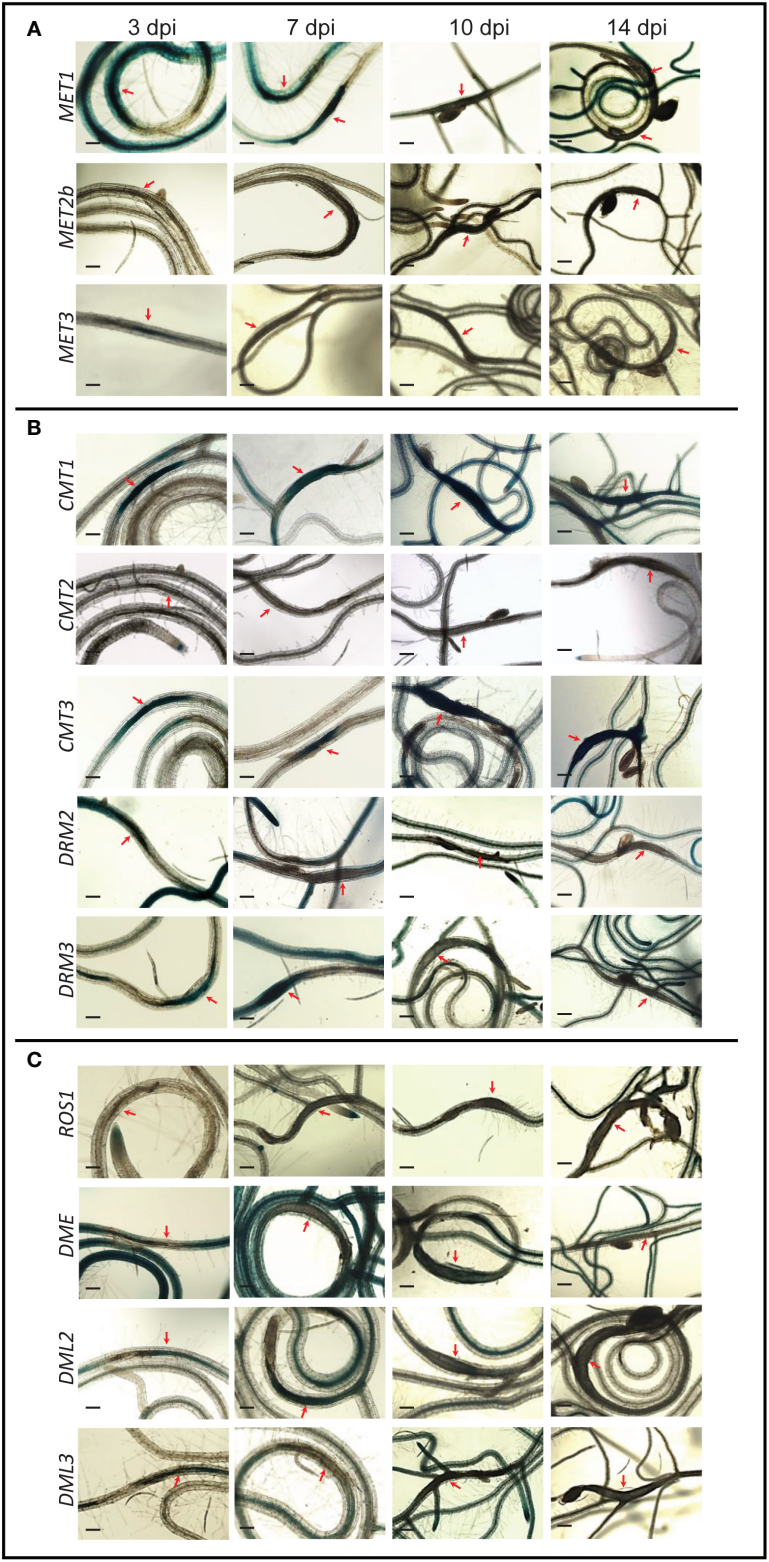
*Promoter activity of DNA methyltransferases and demethylases in the syncytia induced by (*H*)* schachtii *on Arabidopsis roots. Time-course experiments showing GUS activity controlled by the CG methyltransferases* MET1, METb*, and* MET3 **(A)**, *the non-CG methyltransferases* CMT1, CMT2, CMT3, DRM2*, and* DRM3 **(B)**, and the DNA demethylases ROS1, DME, DML2*, and* DML3 **(C)** *at 3, 7, 10, and 14 dpi. Red arrows point to syncytia. Scale bar = 200 µm*.

Seeds of transgenic Arabidopsis lines (T2 generation) expressing the β-glucuronidase (GUS) reporter, guided by the promoter of 12 different DNA methyltransferase and demethylase genes were planted. Ten‐day‐old seedlings were inoculated with second‐stage juvenile (J2) nematodes of *Heterodera schachtii* or *Meloidogyne incognita.* Promoter activity was determined by staining the plants at various time points post inoculation. In response to *H. schachtii* infection, the major CG methyltransferase *MET1* was highly expressed in the syncytia at 3- and 7-day post infection (dpi), as indicated by strong GUS. At later time points, however, no GUS activity directed by *MET1* promoter was detected in the syncytium (**Figure 1A**). The *MET1* homolog *MET3*, whose expression was not detected in roots tissues under non-infected conditions (Bennett et al., 2021), was strongly upregulated in the syncytium at 3 dpi. No activity of *MET2b* promoter was detected in the syncytium at any time points (**Figure 1A**). These expression patterns indicate that maintenance of the CG-context methylation in the syncytia is restricted to early stages of syncytium formation and development.

Promoter activity of *CMT3*, the main CHG methyltransferase gene, and its homolog *CMT1*, revealed similar patterns of high expression in the syncytium at all time points (**Figure 1B**), suggesting that CHG-context methylation is actively maintained in the syncytium during early and late stages of *H. schachtii* infection. In contrast, the expression of *CMT2* was not activated in the syncytium at any time points. The RdDM methyltransferase genes *DRM2* and *DRM3* showed similar expression patterns of upregulation in the syncytia at 3 dpi and downregulation at 14 dpi (**Figure 1B**). However, at 7 and 10 dpi *DRM2* and *DRM3* exhibited opposite expression patterns, i.e., *DRM2* was downregulated and *DRM3* was upregulated in the syncytia (**Figure 1B**). These results suggest that *de novo* DNA methylation is actively established in the syncytia.

The DNA demethylase genes *ROS1, DME*, and *DML3* were downregulated in the syncytia during early and/or late infection stages. Unlike these three DNA demethylases, *DML2* was upregulated in the syncytia at 3 and 7 dpi (**Figure 1C**), suggesting active DNA demethylation in the syncytium is mediated solely through the activity of DML2.

To further confirm promoter activity data, we quantified the expression levels of *MET1*, *MET3*, *DRM2*, *ROS1*, and *DML2* in *H. schachtii-*infected roots of wild-type Col-0 plants using reverse transcription quantitative PCR (RT-qPCR) at 4 dpi. The expression levels of *MET1*, *DRM2*, *ROS1*, and *DML2* were significantly upregulated in infected roots relative to non-infected control plants (**Figure S2**), confirming the increased promoter activity of these genes upon *H. schachtii* infection. However, *MET3*, showed significant downregulation (**Supplementary Figure 2**) despite its promoter was strongly and specifically upregulated in the syncytium, suggesting opposite regulation of *MET3* in roots versus syncytial cells in response to *H. schachtii* infection.

### Mutations of DNA methylation and demethylation-related genes alter plant susceptibility to *Heterodera schachtii*

We next investigated the impact of mutations in these DNA methylation-related genes on plant response to nematode infection. Homozygous T-DNA insertional mutants for 10 genes were identified and phenotypically analyzed. This included mutants of *MET2b* (*SALK_093835C* and *SALK_102231C*), *MET3* (*SALK_099592C* and *SALK_024049C*), *CMT1* (*SALK_138685C* and *SALK_030404C*), *CMT2* (*SALK_012874C* and *CS879822*), *CMT3* (*CS6365*), *DRM2* (*CS16386* and *SALK_129477C*), *DRM3* (*SALK_024820C* and *SALK_136439C*), *ROS1(SALK_045303C* and *CS66099*) *DML2* (*SALK_131712C*), and *DML3* (*SALK_056440C*) (**Supplementary Figure 1**). Homozygous mutants of *MET1* and *DME* are embryonic lethal or associated with severe developmental irregularities (Choi et al, 2002; Kankel et al., 2003; Saze et al., 2003; Mathieu et al, 2007), and therefore were not included in the susceptibility assays. No severe morphological irregularities in the shoots of mutants assessed in this study were detected when compared with the wild-type plants (**Supplementary Figure 3**). With the exception of the short root phenotypes observed in the *ROS1* mutants (*SALK_045303C* and *CS66099*), no noticeable morphological changes in root structure and length were noticed in these mutants as compared with wild-type plants (**Supplementary Figure 4**).

**Figure 2:**
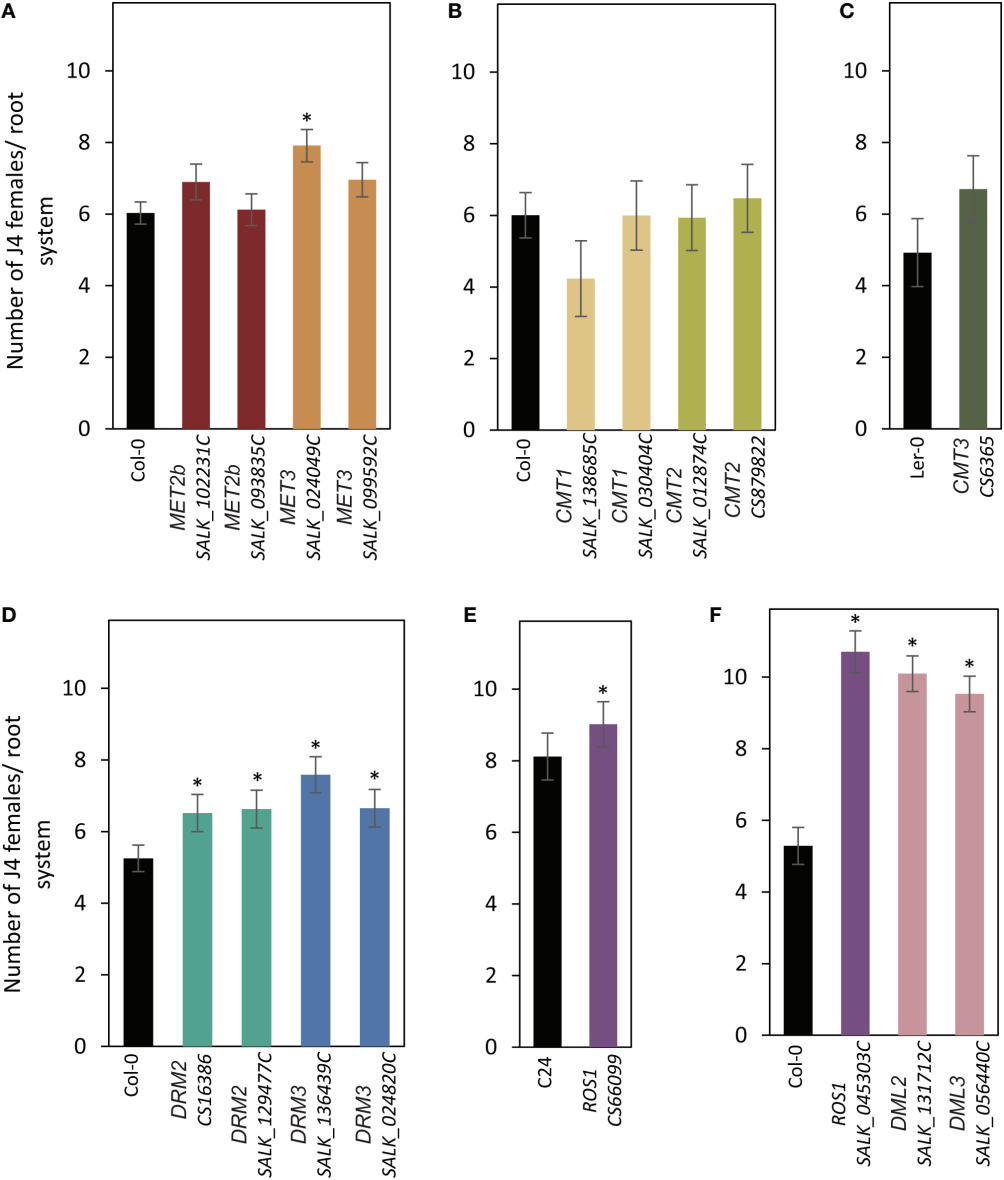
Mutations of DNA methyltransferases and demethylases enhance plant susceptibility to *H. schachtii*. **(A–E)**: *H. schachtii* infection assays of mutant alleles of *MET2b* (*SALK_102231C* and *SALK_093835C*), *MET3* (*SALK_024049C* and *SALK_099592C*) **(A)**, *CMT1* (*SALK_138685C* and *SALK_030404C*) and *CMT2* (*SALK_012874C* and *CS879822*) **(B)**, *CMT3* (*CS6365*) **(C)**, *DRM2* (*CS16386* and *SALK_129477C*) and *DRM3* (*SALK_136439C* and *SALK_024820C*) **(D)**, *ROS1* (*CS66099*) **(E)**, *ROS1* (*SALK_045303C*), *DML2* (*SALK_131712C*), and *DML3* (*SALK_056440C*) **(F)**. The number of J4 female nematodes per root system was counted 3 weeks post inoculation. Data are presented as means ± SE (n = 20). Asterisks denote statistically significant differences from the wild-type Col-0, Ler or C24 as determined by *t* tests (*P* < 0.05).

Seeds of the mutant lines along with the corresponding wild types were planted in 12-well plates and inoculated with freshly hatched J2s of *H. schachtii*. The numbers of nematode J4 females per root system were counted three weeks after inoculation and used as a measurement of plant susceptibility. As shown in **Figure 2A**, both mutant alleles of *MET2b* (*SALK_102231C* and *SALK_093835C*) showed susceptibility levels similar to the wild-type Col-0, consistent with the absence of *MET2b:GUS* activity in the syncytium. In contrast, the *MET3* mutant (*SALK_024049C*) was significantly more susceptible to *H. schachtii* in comparison with the wild-type Col-0 (**Figure 2A**), suggesting that CG hypomethylation is associated with increased plant susceptibility to *H. schachtii*. All mutants of the three *CMT* genes did not show any significant impact on plant susceptibility (**Figures 2B, C**). Interestingly, both mutant alleles of *DRM2* and *DRM3* showed statistically significant increases in plant susceptibility (**Figure 2D**). These results suggest that non-CG hypomethylation is also associated with increased plant susceptibility to *H. schachtii*. All tested mutants of the DNA demethylase genes *ROS1*, *DML2*, and *DML3* were more susceptible as compared with the corresponding wild-type Col-0 or C24 (**Figures 2E, F**), signifying that inhibition of active DNA demethylation promotes *H. schachtii* parasitism of Arabidopsis.

### Promoter activity of DNA methyltransferases and demethylases in the galls induced by the root-knot nematode *Meloidogyne incognita*

**Figure 3:**
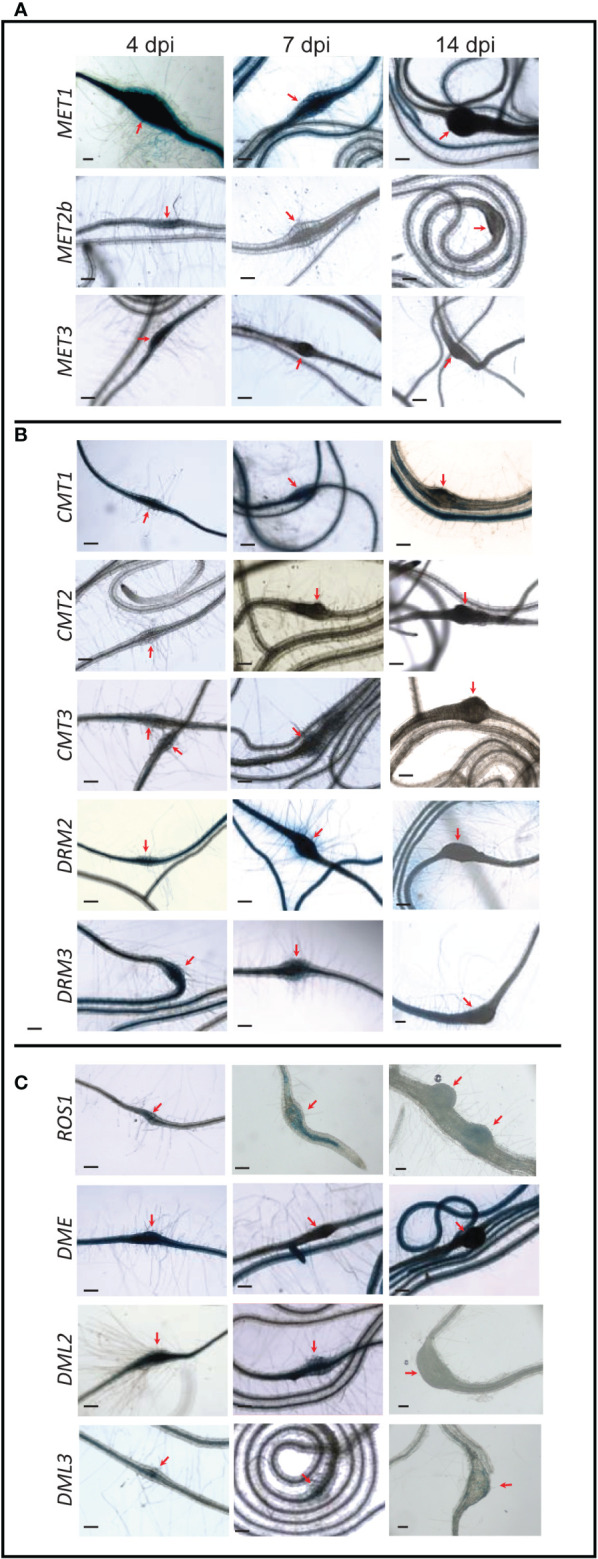
*Promoter activity of DNA methyltransferases and demethylases in the galls induced by* M. incognita *on Arabidopsis roots. Time-course experiments showing GUS activity controlled by the CG methyltransferases* MET1, MET2b*, and* MET3 **(A)**, *the non-CG methyltransferases* CMT1, CMT2, CMT3, DRM2*, and* DRM3 **(B)**, and the DNA demethylases ROS1, DME, DML2*, and* DML3 **(C)** in the galls formed by *M. incognita* at 4, 7, and 14 dpi. Red arrows point to galls. Scale bar = 200 µm.

The CG methyltransferase genes *MET1*, *MET2b*, and *MET3* were expressed in the galls induced by the root-knot nematode *M. incognita* at 4 dpi (**Figure 3A**). At 7 dpi, however, only *MET1* showed GUS staining in the galls. At 14 dpi, none of these three genes was activated in the galls, suggesting that CG methylation is maintained in the gall tissues during the early stage of infection.

The *CMT* gene family members showed varied expression patterns in the galls (**Figure 3B**). While the promoter activity of *CMT1* can be seen in the galls at 4, 7, and 14 dpi, *CMT3* promoter was active in the galls only at 7 dpi. In contrast, GUS activity driven by *CMT2* promoter was not detected in the galls at any time points (**Figure 3B**). These data suggest that variable activities of the enzymes mediating CHG methylation occur in the galls induced by *M. incognita*. *DRM2* and *DRM3* exhibited high expression in the galls at all time points, implying a role for *de novo* DNA methylation in establishing gall methylomes during various parasitic stages.

The expression of *ROS1, DME, DML2*, and *DML3* was observed in the galls at 4 dpi (**Figure 3C**), signifying a role for active DNA demethylation during early stage of *M. incognita* infection. However, at 7 dpi, only *DML2* showed strong GUS staining in the galls (**Figure 3C**). *ROS1* and *DML3* showed weak but visible GUS staining at 7 dpi (**Figure 3C**). At 14 dpi, none of these four DNA demethylase genes showed detectable GUS staining in the galls (**Figure 3C**), suggesting that active DNA demethylation occurs primarily during the early stage of *M. incognita* infection.

Furthermore, the expression levels of *MET1*, *MET3*, *DRM2*, *ROS1*, and *DML2* were measured in *M. incognita-*infected roots of wild-type Col-0 plants at 4 dpi using RT-qPCR to validate the promoter activity of these genes. *MET1*, *MET3*, *ROS1*, and *DML2* showed between 2 and 5-fold upregulation in infected roots compared with non-infected roots (**Figure S5**), consistent with the strong GUS activity of these genes detected after *M. incognita* infection at this time point.

### Mutations of DNA methylation and demethylation-related genes alter plant susceptibility to *Meloidogyne incognita*

**Figure 4:**
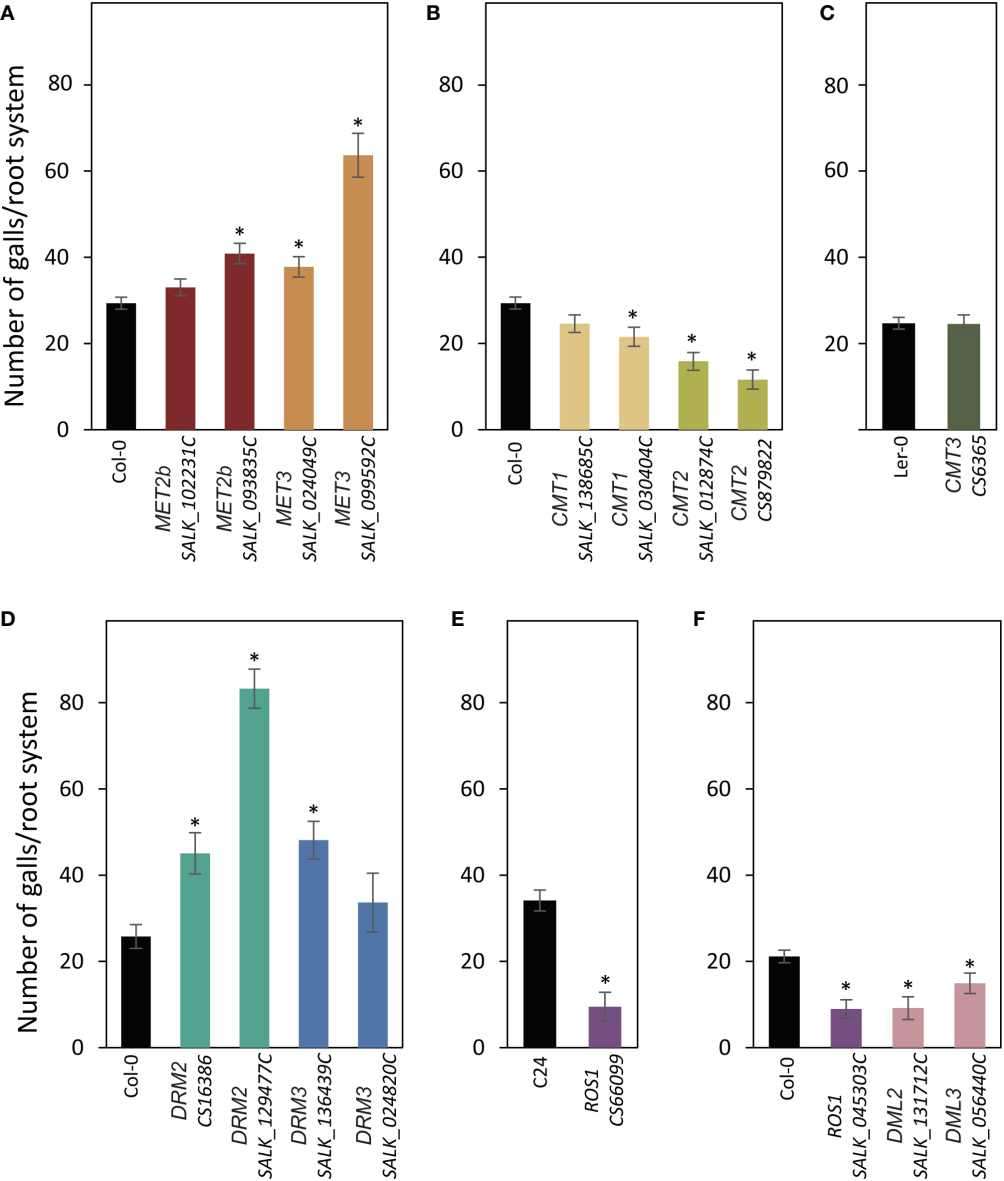
Mutations of DNA methyltransferases and demethylases alter plant susceptibility to *M. incognita.* **(A–E)**: M. incognita infection assays of mutant alleles of *MET2b* (*SALK_102231C* and *SALK_093835C*), *MET3* (*SALK_024049C* and *SALK_099592C*) **(A)**, *CMT1* (*SALK_138685C* and *SALK_030404C*) and *CMT2* (*SALK_012874C* and *CS879822*) **(B)**, *CMT3* (*CS6365*) **(C)**, *DRM2* (*CS16386* and *SALK_129477C*) and *DRM3* (*SALK_136439C* and *SALK_024820C*) **(D)**, *ROS1* (*CS66099*) **(E)**, *ROS1* (*SALK_045303C*), *DML2* (*SALK_131712C*) and *DML3* (*SALK_056440C*) **(F)**. The number of galls per root system was counted 3 weeks post inoculation. Data are presented as means ± SE (n = 20). Asterisks denote statistically significant differences from the wild-type Col-0, Ler or C24 as determined by *t* tests (*P* < 0.05).

The T-DNA insertional mutants mentioned above were also evaluated for their susceptibility to *M. incognita.* Three-week-old plants growing in soil were inoculated with freshly hatched J2s of *M. incognita*, and the number of galls per root system was counted three weeks after inoculation and used to determine susceptibility levels of the mutants in comparison with the corresponding wild-type plants. Of the genes involved in CG methylation, we found that mutant alleles of *MET2b* (*SALK_093835C*) and *MET3* (*SALK_099592C* and *SALK_024049C*) were significantly more susceptible as compared with the wild-type Col-0 plants (**Figure 4A**). In contrast, mutants of *CMT1* (*SALK_138685C*) or *CMT2* (*SALK_012874C* and *CS879822*) were statistically less susceptible to *M. incognita* compared with Col-0 (**Figure 4B**). Susceptibility level of the *CMT3* mutant (*CS6365*) was comparable to that of wild-type Ler-0 (**Figure 4C**). Mutant alleles of *DRM2* (*CS16386* and *SALK_129477C*) and *DRM3* (*SALK_136439C*) showed statistically significant increases in plant susceptibility (**Figure 4D**). These results suggest that genes involved in the CG and non-CG methylation may exert opposite effects on plant susceptibility to *M. incognita.* Interestingly, all examined mutants of the DNA demethylases *ROS1* (*SALK_045303C* and *CS66099*), *DML2* (*SALK_131712C*), and *DML3* (*SALK_056440C*) were statistically less susceptible to *M. incognita* as compared with the corresponding wild-type Col-0 or C24 (**Figures 4E, F**), implying the importance of active DNA demethylation for *M. incognita* parasitism of Arabidopsis.

### Defense response genes are oppositely regulated in DNA demethylase mutants in response to infection by cyst and root-knot nematodes

**Figure 5:**
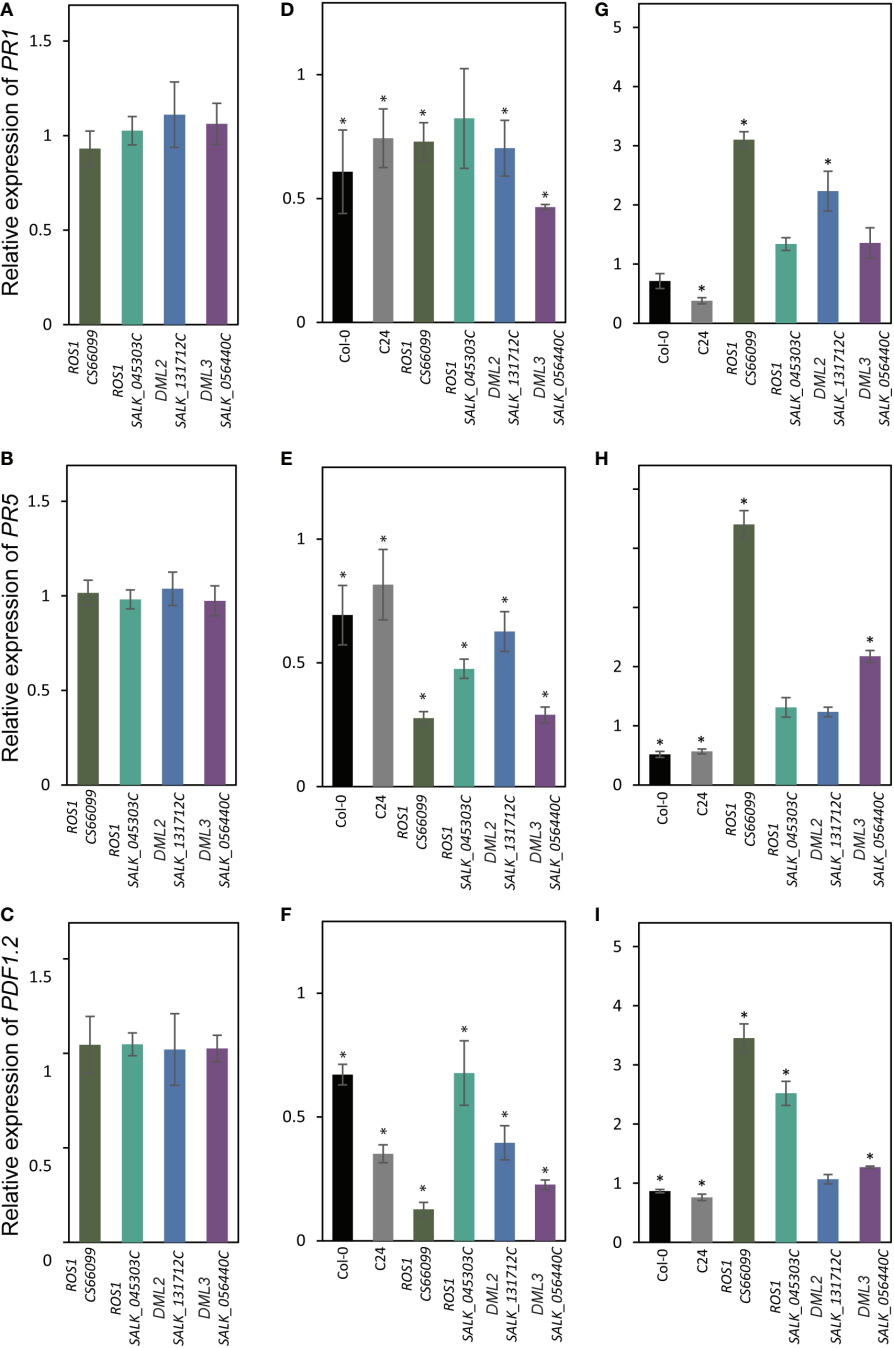
Expression levels of PR *genes in DNA demethylase mutants* in response to infection by cyst and root-knot nematodes. **(A–C)**: RT-qPCR quantification of the expression of PR1 **(A)**, PR5 ) and PDF1.2 **(C)** in roots tissues of mutant alleles of *ROS1* (*CS66099* and *SALK_045303C*), *DML2* (*SALK_131712C*), and *DML3* (*SALK_056440C*) under non-infected conditions relative to wild-type plants. **(D-F)**: Expression of PR1 **(D)**, PR5 **(E)** and PDF1.2 **(F)** in the **(H)** schachtii-infected roots tissues of wild-type (Col-0 and C24) and mutant alleles of *ROS1* (*CS66099* and *SALK_045303C*), *DML2* (*SALK_131712C*), and *DML3* (*SALK_056440C*) relative to non-infected root tissues. **(G–I)**: Expression of PR1 **(G)**, PR5 **(H)** and PDF1.2 **(I)** in the M. incognita-infected roots tissues of wild-type (Col-0 and C24) and mutant alleles of *ROS1* (*CS66099* and *SALK_045303C*), *DML2* (*SALK_131712C*), and *DML3* (*SALK_056440C*) relative to non-infected root tissues. Relative gene expression levels were obtained from three biological samples and presented as mean ± SE. *PP2AA3* and *actin8* were used as internal reference genes to normalize gene expression. Asterisks indicate statistically significance differences from control treatments at P < 0.05.

Several reports have indicated that DNA hypomethylation contributes to the regulatory mechanism of plant-induced defense responses (Yu et al., 2013; Le et al., 2014; Lopez Sanchez et al., 2016). Therefore, we examined the expression levels of pathogenesis related (*PR*) genes in the DNA demethylase mutants showing contrasting susceptibility phenotypes in response to infection by *H. schachtii* and *M. incognita*. We first quantified the expression of *PR1*, *PR5*, and *PLANT DEFENSIN1.2* (*PDF1.2*) in the root tissues of *ros1* (*CS66099* and *SALK_045303C*), *dml2* (*SALK_131712C*) and *dml3* (*SALK_056440C*) under non-infected conditions. As shown in **Figures 5A–C**, *PR1*, *PR5* and *PDF1.2* expression was comparable to that of the wild-type plants Col-0 or C24.

We next quantified the expression of *PR1*, *PR5*, and *PDF1.2* in the root tissues of these four DNA demethylase mutants and wild-type plants (Col-0 and C24) infected with *H. schachtii* or *M. incognita* at 4 dpi. In response to *H. schachtii* infection, the expression of these genes showed significant downregulation in infected wild-type roots compared with non-infected roots (**Figures 5D–F**). Similarly, the expression levels of *PR1*, *PR5*, and *PDF1.2* were significantly downregulated in almost all *H. schachtii*-infected mutant lines when compared with non-infected mutants (**Figures 5D–F**). However, the levels of downregulation of *PR5* and *PDF1.2* were much higher in the infected mutants than the infected wild types (**Figures 5E, F**).

In response to *M. incognita* infection, the expression of *PR1*, *PR5*, and *PDF1.2* was downregulated in the infected wild-type roots versus non-infected roots (**Figures 5G–I**). In the mutant lines, however, we observed a general trend of upregulation of the *PR1*, *PR5*, and *PDF1.2* transcripts in *M. incognita*-infected roots versus non-infected roots (**Figures 5G–I**). Together, these results suggest that opposite regulation of defense-related genes in the mutant lines in response to infection by *H. schachtii* and *M. incognita* may have contributed to the observed opposite susceptibility phenotypes.

## Discussion

Our analysis revealed an important role of DNA methylation and active demethylation pathways in mediating Arabidopsis interactions with *H. schachtii* and *M. incognita*. Promoter activity and mutant analyses of 12 key genes involved in DNA methylation or active demethylation unveiled important similarity and distinct differences between these two pathosystems in term of expression patterns and plant susceptibility. For example, the promoter activity of *MET1* and its homolog *MET3* were both detected in the syncytia and galls during the early stage of infection. In contrast, the expression of *MET2b* was detected only in galls at 4 dpi (**Figures 1A**, **3A**), a finding that is consistent with increased susceptibility of *met2b* mutant plants *(SALK_093835C*) to *M. incognita* but not *H. schachtii* (**Figures 2B**, **4B**). Strikingly, no such activity of *MET3* promoter was detected in the root tissues under non-infected conditions (Bennett et al., 2021), but this promoter was activated specifically in the syncytia and galls only at 3 and 4 dpi, respectively (**Figures 1A**, **3A**). Consistent with this finding, mutant alleles of *MET3* were more susceptible to both *H. schachtii* and *M. incognita* (**Figures 2B**, **4B**). The finding that *MET3* expression is restricted to the developing embryo (Jullien et al., 2012; Bennett et al., 2021) suggests a unique role of the encoding enzyme in reprogramming CG methylation in the syncytia and galls in a way similar to the developing embryos, which undergo extensive DNA methylation changes (Xiao et al., 2006; Gehring et al., 2009; Jullien et al., 2012; Wang et al., 2015; Satyaki and Gehring, 2017).

Notably, none of the examined mutants of the *CMT* genes were found to alter plant susceptibility to *H. schachtii* despite the strong activation of *CMT1* and *CMT3* in the syncytia during both early and late stages of infection. While this finding can be attributed to a possible functional redundancy between CMT1 and CMT3, whose expression was detected in the syncytia (**Figure 1B**), this explanation doesn’t support the results showing that single mutants of this gene family were altered in their susceptibility to *M. incognita* (**Figures 4B, C**). One possible explanation is that global loss of CHG methylation in *cmt1* and *cmt3* (Stroud et al., 2013) mutants have little or no effect on syncytium formation and function. Alternatively, global decrease in CHG methylation can be compensated with an increase of CG and CHH methylation in the syncytium as recently reported (Rambani et al., 2020b). In support of this hypothesis, the expression of *MET1* and *MET2b* as well as *DRM2* and *DRM3*, which establish CHH methylation *de novo*, are strongly induced in the syncytia (**Figures 1A-B**). Although DRMs are established as CHH methyltransferases, they regulate CHG methylation as well (Cao and Jacobsen, 2002b; Stroud et al., 2013).

The intense GUS staining of the *DRM2* and *DRM3* promoters in the syncytia and galls (**Figures 1B**, **3B**) along with the results showing that mutant alleles of *DRM2* and *DRM3* were more susceptible to both *M. incognita* and *H. schachtii* point to the importance of establishing DNA methylation *de novo* in syncytial and gall cells as a defense mechanism to control cyst and root-knot nematode infection. Similarly, RdDM-defective mutants were found to be more susceptible to *Botrytis cinerea* and *Plectosphaerella cucumerina* (López et al., 2011). More recently, it was shown that tomato *DRM5* expression is significantly upregulated in the roots of a resistant tomato cultivar upon infection with a virulent root-knot nematode field population (Leonetti and Molinari, 2020). However, various rice mutants deficient in the RdDM pathway were reported less susceptible to *M. graminicola* despite the fact that more than 99% of the identified differentially methylated regions in the galls were CHH-hypomethylated (Atighi et al., 2020). Similarly, it has been recently shown that *M. javanica* reproduction on *drm1/drm2* double mutant was greatly reduced (Silva et al., 2022). Together, these findings suggest that the RdDM pathway regulates plant resistance against various phytopathogens (Erdmann and Picard, 2020) including cyst and root-knot nematodes.

It may be important to mention that the differences between the two alleles of *MET3* (*SALK_099592C* and *SALK_024049C*), *CMT1* (*SALK_138685C* and *SALK_030404C*), and *DRM3* (*SALK_136439C* and *SALK_024820C*) in response to infection by *H. schachtii* or *M. incognita* are most likely due to difference in gene expression as shown in **Supplementary Figure 1B**. Similarly, of the two mutant lines of *MET2b* (SALK_102231 and SALK_093835C) only SALK_093835C exhibited increased susceptibility to *M. incognita* despite the fact that *MET2b* is expressed at a similar level in both mutants. The differences between the two mutant alleles could be related to different T-DNA insertion sites (**Supplementary Figure 1A**), which may impact the function of truncated transcripts.

Remarkably, the promoter activity of *ROS1, DME, DML2*, and *DML3* exhibited opposite expression patterns in the syncytia and galls (**Figures 1C**, **3C**). With the exception of *DML2* upregulation in the syncytium at 3 and 7 dpi, the remaining three DNA demethylase genes were either downregulated or not expressed at a detectable level in the syncytia (**Figure 1C**). In contrast, strong GUS activity was observed for all four demethylase genes in young galls at 4 dpi (**Figure 3C**). Consistent with the contrasting expression patterns, the mutant alleles of *ROS1, DML2*, and *DML3* showed opposite responses to infection by cyst and root-knot nematodes. Indeed, the DNA demethylase mutants exhibited increased susceptibility to *H. schachtii* and decreased susceptibility to *M. incognita* (**Figures 2C**, **3C**). The involvement of active DNA demethylases in disease resistance has been reported in few studies. For example, a loss-of-function mutation in the Arabidopsis *ROS1* gene enhanced plant susceptibility to the bacterial pathogen Pseudomonas syringae *pv. tomato DC3000* (*Pto DC3000*) (Yu et al, 2013). In addition, Arabidopsis *ros1/dml2/dml3 (rdd)* triple mutant displayed enhanced disease susceptibility to the fungal pathogen Fusarium oxysporum (Le et al., 2014). The opposite response of Arabidopsis hypermethylated mutants to various plant pathogens has also been reported. For instance, the hypermethylated mutant *ros1* was less resistant to *Hpa*, but more resistant to the necrotrophic fungi *Alternaria brassicicola* and *Plectosphaerella cucumerina* as well as *M. javanica* (López Sánchez et al., 2016; Silva et al., 2022).

Our gene expression analysis of *PR1*, *PR2*, and *PDF1.2* pointed to opposite regulation of defense-related genes in the DNA demethylase mutants in response to infection by *M. incognita* and *H. schachtii* (**Figures 5B, C**). This may partially explain the opposite susceptibility phenotypes of these mutants in response to infection by *H. schachtii* and *M. incognita* (**Figures 2C**, **4C**), and highlights the complexity of plant defense pathways against plant-parasitic nematodes and their interactions with active demethylation pathway. Our results are consistent with the finding that active DNA demethylases function mainly in regulating the expression of defense- and stress-related genes (Yu et al., 2013 ***;*** Le et al., 2014 ***;*** Lopez Sanchez et al., 2016) and point to distinct methylation-dependent mechanisms regulating defense gene expression upon infection by cyst and root-knot nematodes. Basal defense response does not seem to be altered in the demethylase mutants under non-infected conditions because *PR1*, *PR2*, and *PDF1.2* are similarly expressed in these mutants and wild-type plants (**Figures 5A–C**). Additionally, it is unlikely that changes in the expression of *PR1*, *PR5*, and *PDF1.2* are due to changes in methylation status in *cis* at the promoters of these genes upon nematode infection because nematode-induced potential hypermethylation of these *PR* genes in four demethylated mutants argues against upregulation of these genes in response to *M. incognita* infection (**Figures 5G–I**). Furthermore, *PR1*, *PR2*, and *PDF1.2* are not subjected to DNA methylation changes upon *H. schachtii* infection in Col-0 plants (Hewezi et al., 2017). Additionally, it has been shown that the majority of defense-related genes in *ros1* mutant are indirectly regulated by DNA methylation (López Sánchez et al., 2016; Halter et al., 2021). Taken together, it is conceivable to suggest that changes in DNA methylation patterns of *trans*-acting factors directly or indirectly controlling the expression of defense- and stress-related genes in DNA demethylase mutants are the causal factors contributing towards opposite regulation of *PR1*, *PR2*, and *PDF1.2* upon infection by *H. schachtii* and *M. incognita.* Taken together, our results set the stage for future studies to determine the exact mechanism through which DNA methylation and active demethylation of *trans*-acting factors modulate plant immunity and basal defense response upon infection with parasitic nematodes. This may eventually lead to the development of novel approaches to improve plant resistance to nematode infection.

## Data availability statement

The original contributions presented in the study are included in the article/**Supplementary Material**. Further inquiries can be directed to the corresponding author.

## Author contributions

TH designed the original research plans. MB performed the majority of the experimental work, analyzed the data, and wrote the first draft. TEH, VL-C, NA, and JR helped MB in data collection and analysis. TH supervised the experimental work, analyzed the data, and wrote the final version. All authors contributed to the article and approved the submitted version.
