# Supplementary material for: Establishment and maintenance of DNA methylation in nematode feeding sites
Source: Front Plant Sci. 2023 Jan 10;13:1111623. doi: 10.3389/fpls.2022.1111623 (PMC9873351; doi:10.3389/fpls.2022.1111623)
Supplement: Supplementary file 2 [file DataSheet_1.pdf]

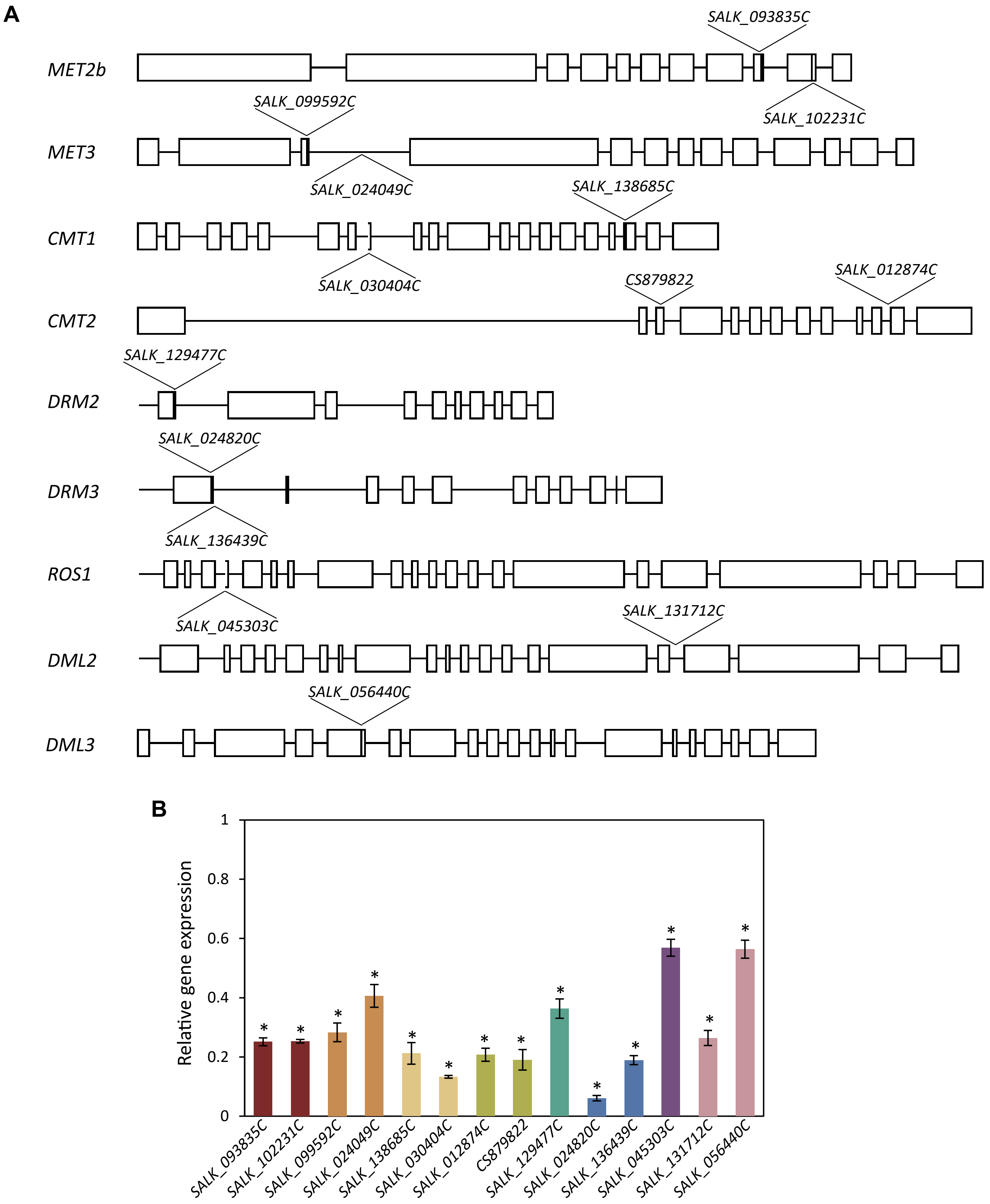

Supplemental Figure 1: Characterization of Arabidopsis DNA methyltransferase and demethylase mutants.
A: Schematic representation of various DNA methyltransferase and demethylase mutant alleles showing locations of the T-DNA insertions. Exons and introns are represented by boxes and lines, respectively.
B: mRNA expression levels in various Arabidopsis DNA methyltransferase and demethylase mutants. mRNA expression levels were determined in 10-day-old plants using RT-qPCR. *PP2AA3* and *actin8* were used as internal reference genes to normalize gene expression levels. Relative fold-change values reflect changes in mRNA abundance in the mutant lines relative to wild-type plants. Shown are averages of three biologically independent samples ± SE. Asterisks indicate statistically significance differences from the wild-type plants at $P < 0.05$.
