# Supplementary material for: Establishment and maintenance of DNA methylation in nematode feeding sites
Source: Front Plant Sci. 2023 Jan 10;13:1111623. doi: 10.3389/fpls.2022.1111623 (PMC9873351; doi:10.3389/fpls.2022.1111623)
Supplement: Supplementary file 3 [file DataSheet_2.pdf]

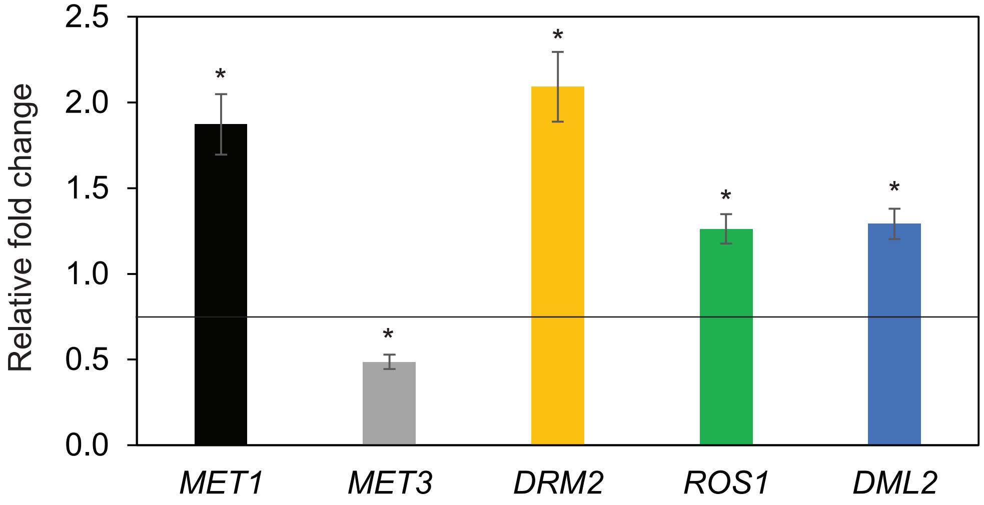

Supplemental Figure 2: Expression levels of *MET1, MET3, DRM2, ROS1,* and *DML2* in root tissues of Col-0 plants in response to *H. schachtii* infection at 4 dpi.

The expression levels of *MET1, MET3, DRM2, ROS1*, and *DML2* were quantified in root tissues of wild-type Col-0 plants using three biological samples each with two technical replicates. Relative fold change values represent changes in *H. schachtii*-infected root samples relative to non-infected control samples, which were set to 1. *Actin 8* and *PP2AA3* were used as internal reference genes to normalize gene expression levels. Asterisks denote statistically significant differences between *H. schachtii*-infected and non-infected root samples at $P < 0.05$ using *t*-test.
