# Supplementary material for: Establishment and maintenance of DNA methylation in nematode feeding sites
Source: Front Plant Sci. 2023 Jan 10;13:1111623. doi: 10.3389/fpls.2022.1111623 (PMC9873351; doi:10.3389/fpls.2022.1111623)
Supplement: Supplementary file 4 [file DataSheet_3.pdf]

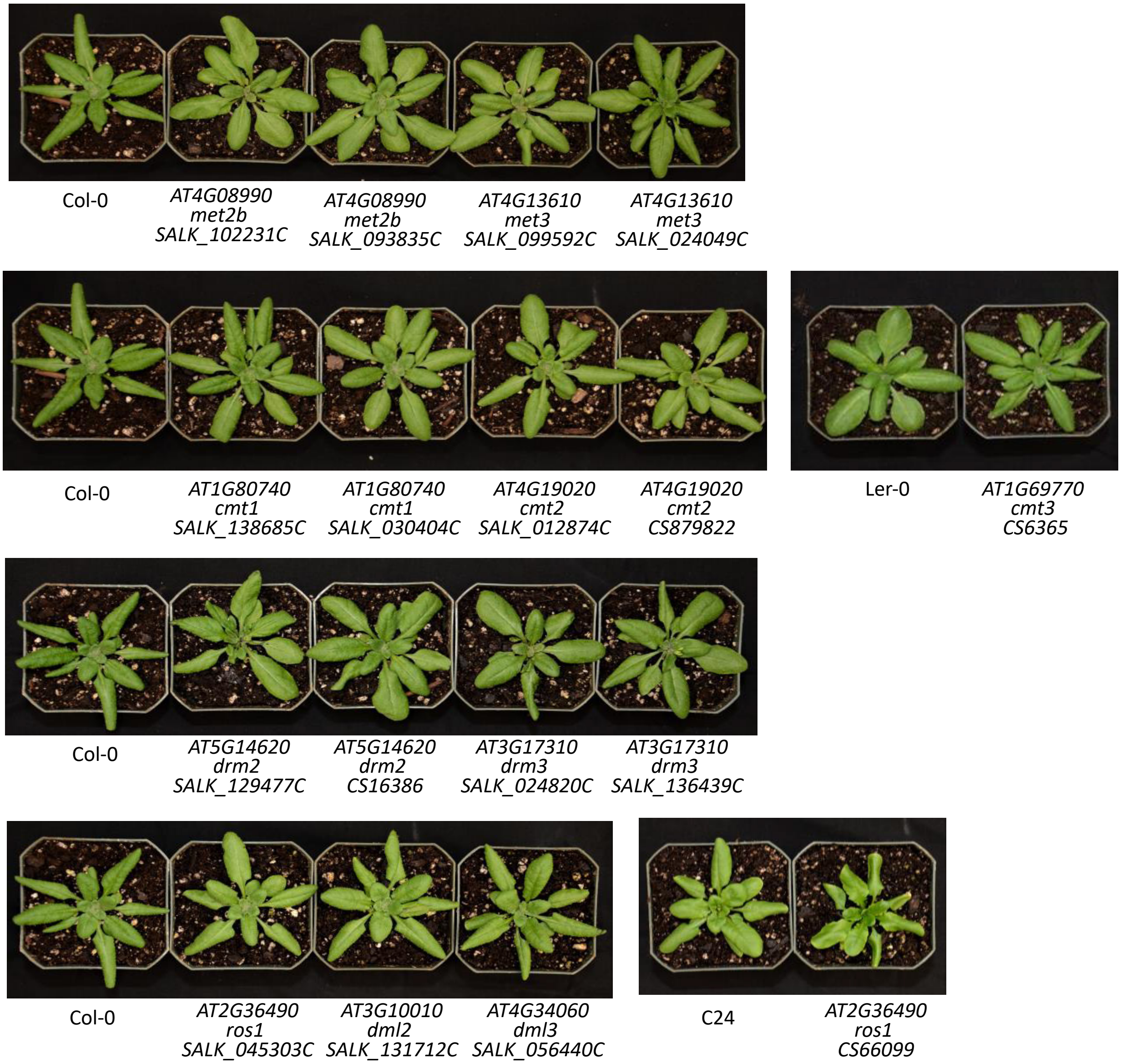

Supplemental Figure 3: Shoot phenotypes of four-week-old Arabidopsis DNA methyltransferase and demethylase mutants used in the current study.
Seeds of the indicated mutant lines and wild types were planted in soil and shoot phenotypes of four-week-old plants were photographed.
