# Supplementary material for: Establishment and maintenance of DNA methylation in nematode feeding sites
Source: Front Plant Sci. 2023 Jan 10;13:1111623. doi: 10.3389/fpls.2022.1111623 (PMC9873351; doi:10.3389/fpls.2022.1111623)
Supplement: Supplementary file 5 [file DataSheet_4.pdf]

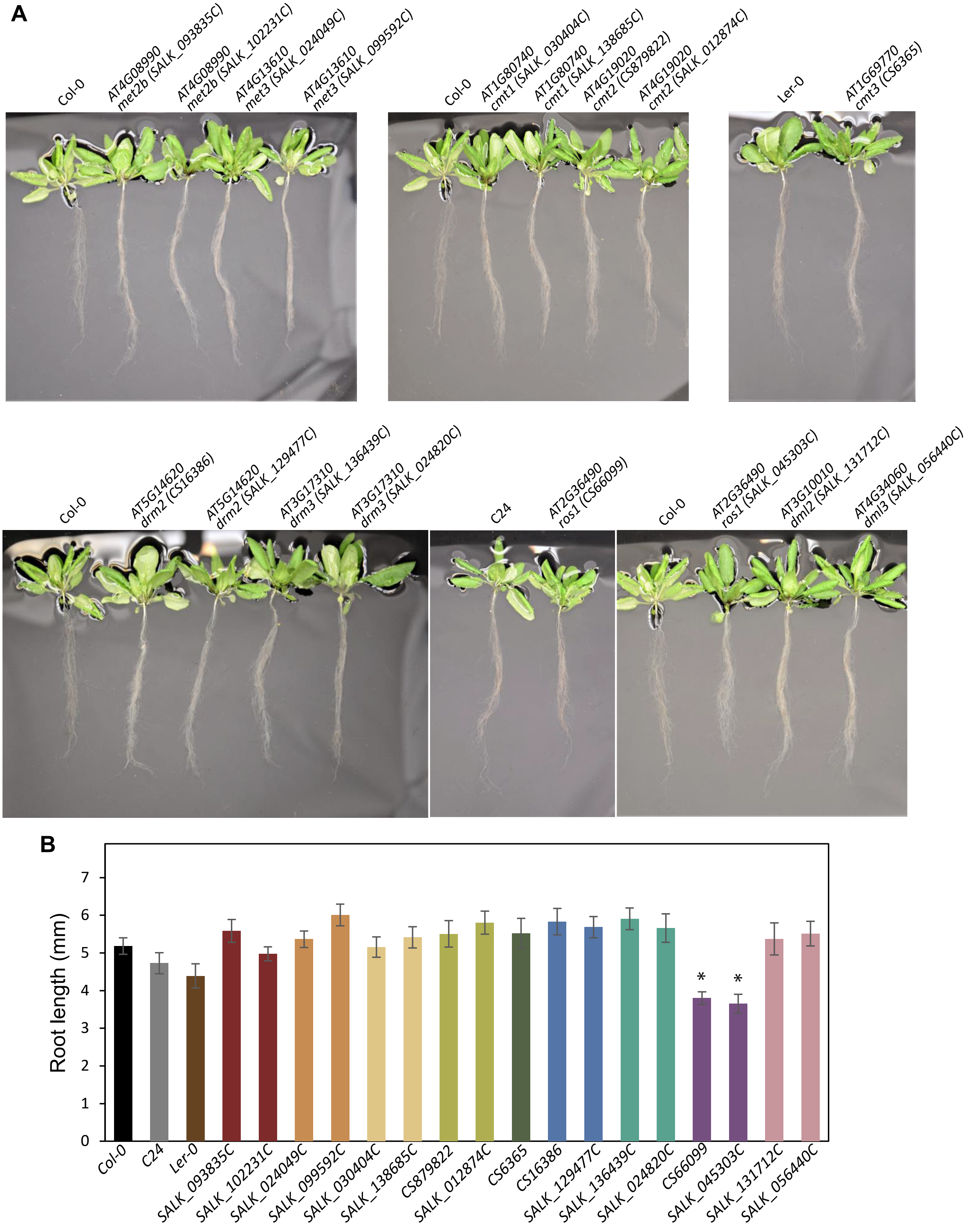

Supplemental Figure 4: Root phenotypes of four-week-old Arabidopsis DNA methyltransferase and demethylase mutants used in this study.

A: Root phenotypes of the indicated four-week-old Arabidopsis DNA methyltransferase and demethylase mutants and the corresponding wild types.

B: Root lengths of the indicated Arabidopsis DNA methyltransferase and demethylase mutants and the corresponding wild types. Root lengths of at least 15 plants were measured two weeks post planting. Data are shown as means ± SE. Root lengths of mutant lines that are statistically significant different ($P < 0.05$) from wild-type plants are indicated by asterisks.
