# Supplementary material for: Establishment and maintenance of DNA methylation in nematode feeding sites
Source: Front Plant Sci. 2023 Jan 10;13:1111623. doi: 10.3389/fpls.2022.1111623 (PMC9873351; doi:10.3389/fpls.2022.1111623)
Supplement: Supplementary file 6 [file DataSheet_5.pdf]

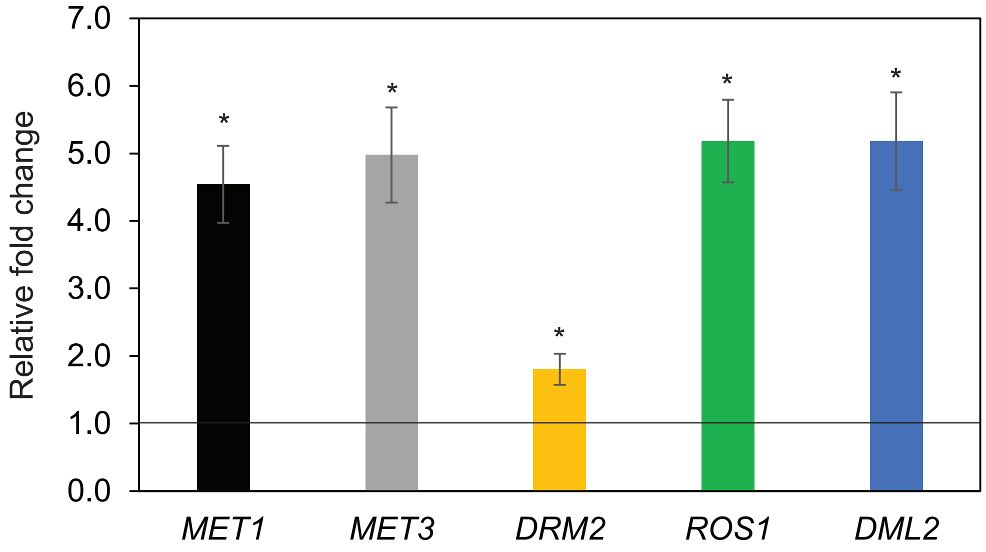

Supplemental Figure 5: Expression levels of *MET1, MET3, DRM2, ROS1,* and *DML2* in root tissues of Col-0 plants in response to *M. incognita* infection at 4 dpi.
The expression levels of M*ET1, MET3, DRM2, ROS1*, and *DML2* were quantified in the root tissues of wild-type Col-0 plants using three biological samples each with two technical replicates. Relative fold change values represent changes in *M. incognita* -infected root samples relative to non-infected control samples, which were set to 1. *Actin 8* and *PP2AA3* were used as internal reference genes to normalize gene expression levels. Asterisks denote statistically significant differences between *M. incognita*-infected and non-infected root samples at $P < 0.05$ using *t*-test.
